# Abstract of Letter from Dr. Sajous to Dr. Gaston

**Published:** 1897-10

**Authors:** Chas. Eden Sajous


### ABSTRACT OF LETTER FROM DR. SAJOUS TO DR. GASTON.

It will be regretted that the Annual of Universal Medical Sciences will no longer appear as such. After detailing the circumstances calling for a different form of publication, he says:

"The great drawback with Annuals, in general, is that they can but present what the year has produced; some diseases are not touched upon and others are merely alluded to, and when a practitioner particularly wishes assistance, it generally happens that none is afforded.

"The new work will present the following features:

"Each article is presented *in toto* as it would be in a text-book, the 'Annual' feature being preserved by the introduction each year of the progressive 'Points' made during that year and obtained from the current literature as heretofore, the general text being modified yearly or oftener, as required.

"Taking the last ten years of literature as presented in the 'Annual,' I selected all such points—the points that in some way contributed something negative or positive upon the etiology, pathology, treatment, etc., of the disease—and grouped them under their respective heads. You will notice that many valuable remarks are thus collected which by intercomparison, may become very suggestive. Four other advantages are thus obtained:

"(1.) The reader is able to compare the various ideas presented and can judge for himself. (2.) He gets a correct view of the exact status of the disease upon which he seeks information. (3.) He has before him not only the most recent methods of treatment which may be valueless, but also the older methods which may be preferable. (4.) The suffrage of the profession at large is placed before him, and he is thus afforded the highest opportunities to make his labors tell at the bedside.

"The proof I send you represents the said material on 'Aneurisms.' As you will notice the article is on a subject other than your own. There being no Thoracic Surgery in the first volume, your valued name would not appear in the latter did you not edit a subject coming under the letters A. or B.

"It enables me to satisfy a wish that your name should be in the first volume, which will, I think, attract great attention. If you will consent to favor me in this manner, I shall be very grateful. Should you desire an assistant, I shall see that he gets a complete set of the work. When completed it will represent about fifteen volumes of the present Annual.

"If you could give it your early attention, you would add another obligation to the many already incurred.

"With kindest regards, I remain,

"Yours very sincerely,

CHAS. EDEN SAJOUS."

